# A randomized clinical trial to determine the effectiveness of CO-oximetry and anti-smoking brief advice in a cohort of kidney transplant patients who smoke: study protocol for a randomized controlled trial

**DOI:** 10.1186/s13063-016-1311-7

**Published:** 2016-04-01

**Authors:** Salvador Pita-Fernández, Rocío Seijo-Bestilleiro, Sonia Pértega-Díaz, Ángel Alonso-Hernández, Constantino Fernández-Rivera, Mercedes Cao-López, Teresa Seoane-Pillado, Beatriz López-Calviño, Cristina González-Martín, Francisco Valdés-Cañedo

**Affiliations:** Clinical Epidemiology and Biostatistics Unit, Instituto de Investigación Biomédica de A Coruña (INIBIC), Complexo Hospitalario Universitario de A Coruña (CHUAC), SERGAS, Universidade da Coruña, Hotel de Pacientes, 7a, As Xubias, 84, 15006 A Coruña, Spain; Nephrology Department, Instituto de Investigación Biomédica de A Coruña (INIBIC), Complexo Hospitalario Universitario de A Coruña (CHUAC), SERGAS, Universidade da Coruña, A Coruña, Spain; Clinical Epidemiology Research Group, Health Sciences Department, Escuela Universitaria de Enfermería y Podología, Universidade da Coruña (UDC), Campus de Ferrol, 15471 Ferrol, Spain

**Keywords:** Smoking cessation, Carbon monoxide, Nicotine dependence, Kidney transplantation

## Abstract

**Background:**

The cardiovascular risk in renal transplant patients is increased in patients who continue to smoke after transplantation.

The aim of the study is to measure the effectiveness of exhaled carbon monoxide (CO) measurement plus brief advisory sessions, in comparison to brief advice, to reduce smoking exposure and smoking behavior in kidney transplant recipients who smoke. The effectiveness will be measured by: (1) abandonment of smoking, (2) increase in motivation to stop smoking, and (3) reduction in the number of cigarettes smoked per day.

**Methods/design:**

Design: a randomized, controlled, open clinical trial with blinded evaluation.

Scope: A Coruña Hospital (Spain), reference to renal transplantation in the period 2012–2015.

Inclusion criteria: renal transplant patients who smoke in the precontemplation, contemplation or preparation stages according to the Prochaska and DiClemente’s Stages of Change model, and who give their consent to participate.

Exclusion criteria: smokers attempting to stop smoking, patients with terminal illness or mental disability that prevents them from participating.

Randomization: patients will be randomized to the control group (brief advisory session) or the intervention group (brief advisory session plus measuring exhaled CO). The sample target size is *n* = 112, with 56 patients in each group. Allowing for up to 10 % loss to follow-up, this would provide 80 % power to detect a 13 % difference in attempting to give up smoking outcomes at a two-tailed significance level of 5 %. Measurements: sociodemographic characteristics, cardiovascular risk factors, treatment, rejection episodes, infections, self-reported smoking habit, drug use, level of dependence (the Fagerström test), stage of change (Prochaska and DiClemente’s Stages of Change model), and motivation to giving up smoking (the Richmond test).

Response: the effectiveness will be evaluated every 3, 6, 9 and 12 months as: pattern of tobacco use (self-reported tobacco use), smoking cessation rates, carbon monoxide (CO) levels in exhaled air measured by CO-oximetry, urinary cotinine tests, nicotine dependence (Fagerström test), motivational stages of change (Prochaska and DiClemente’s stages) and motivation to stop smoking (the Richmond test).

Analysis: descriptive statistics and linear/logistic multiple regression models will be performed. Clinical relevance will be measured as relative risk reduction, absolute risk reduction and the number needed to treat.

Ethics: informed consent of the patients and Ethical Review Board was obtained (code 2011/061).

**Discussion:**

Tobacco is a modifiable risk factor that increase the risk of morbidity and mortality in kidney transplant recipients. If effectiveness of CO-oximetry is confirmed to reduce tobacco exposure, we would have an intervention that is easy to use, low cost and with great implications about cardiovascular risk prevention in these patients.

**Trial registration:**

Current Controlled Trials ISRCTN16615772.

EudraCT number: 2015-002009-12.

## Background

Cigarette smoking is a well-established risk factor for cardiovascular disease and cancer [1]. An adverse effect of smoking on renal function has also been described. Although the exact mechanisms of smoking-induced renal damage remain to be determined, there is clinical evidence that smoking has important adverse effects on renal outcome in various kidney diseases, such as diabetic nephropathy, hypertensive kidney disease, and primary glomerular diseases, as well as for patients who require hemodialysis chronically [2–4]. In renal transplant recipients, who already have a higher risk of cancer [5, 6] and cardiovascular disease [7] than the general population, smoking should probably be viewed as an even more risky behavior.

Despite its well-known harmful effects, smoking prevalence is still high, predominantly in developing countries. The latest World Health Organization (WHO) population survey demonstrated a 29 % rate of smoking in adults in Europe [8]. In Spain, the 2006 National Health Survey (NHS) estimated a 29.5 % smoking rate in adults aged 16 and over [9], with approximately 53,155 smoking-attributable deaths in the same year [10]. Smoking prevalence among kidney transplant recipients is more difficult to estimate, and varies greatly across studies [11]. In a previous work, we found that up to 41.7 % of the renal transplanted patients in our center were smokers at the time of transplantation, and about 15 % continued smoking after the transplant [12]. Although this figure is not very high, the impact of smoking habit on the probability of cardiovascular events in the follow-up of kidney transplant recipients is clinically relevant. As stated in a previous paper, at the 5-year and 10-year follow-up, the number of patients needed to treat (NNT) who would have to give up smoking to prevent a cardiovascular event was 7 and 4, respectively [13].

A recent review [11] identified 12 studies in the period 1968–2009 reporting the effect of cigarette smoking on kidney allograft or recipient survival [14–25]. Although some of the revised studies showed contradictory results, in general cigarette smoking was associated with an increased risk of both graft loss and death. More recently, a large-scale study investigated the effect of smoking on post-kidney transplant outcomes in a retrospective cohort of 41,705 adult renal transplant recipients in the United States Renal Data System [26]. That work concluded that, compared with patients who have never smoked, new onset smoking after transplantation is associated with reduced allograft and patient survival. Similarly, a prospective study of 604 renal transplant recipients in The Netherlands showed that smoking after renal transplantation increases the risk of graft failure and mortality, while past smoking is a risk factor for mortality but not for graft failure [27]. Another European recent study in 402 randomly selected kidney graft recipients also concluded that smoking is a risk factor for kidney damage following renal transplantation [28].

Although there is evidence that active smoking plays an important role in allograft loss and patient mortality, this information has so far had little impact on patient management. Based on these results, smoking cessation should be promoted among patients undergoing kidney transplantation.

Both pharmacotherapy and non-pharmacological interventions could be used for the treatment of tobacco dependence. Non-pharmacological methods have the advantage of being inexpensive and feasible to implement, and may be particularly appropriate for transplant patients, who are usually polymedicated. The main non-pharmacological interventions for the treatment of tobacco dependence include advice from health professionals, self-help material, proactive telephone counseling, group or individual counseling, and intra-treatment or extra-treatment social support.

Although smoking cessation advice from a health professional improves smoking cessation rates, it is only moderately effective, with a smoking cessation rate only 1 to 3 % higher [29]. An alternative to increase smoking cessation rates could be to provide the patients with feedback on the physical effects of smoking via physiological measurements, such as measurement of carbon monoxide (CO) levels in exhaled air by CO-oximetry. Although a recent review could not demonstrate a significant benefit of interventions based on biomedical risk assessments, there is still insufficient evidence regarding this issue [30]. Furthermore, there is scarce data exploring the effect of these kinds of interventions on hospitalized patients or acutely ill patients. It is possible that such a specific context and the presence of co-existent illnesses could facilitate a modification of risk perception.

Currently, different projects are trying to determine the efficacy of adding spirometry or CO-oximetry information to brief advice on smoking cessation, in the primary health care setting [31, 32]. Measuring the level of CO in smokers’ exhaled air can motivate them to stop smoking or be a useful tool in monitoring their progress towards cessation. We aim to test its efficiency in a more restricted setting, studying its utility to promote smoking cessation among kidney transplanted patients. The objective of this study is to measure the effectiveness of exhaled CO plus brief advisory sessions, in comparison to brief advice, to reduce smoking exposure and smoking behavior in kidney transplant recipients who smoke. The effectiveness will be measured by:

1. Self-reported tobacco use: abandonment of smoking habit and change in the number of cigarettes per day smoked
2. Abandonment of smoking habit confirmed through a urinary cotinine test
3. Variation in the motivational stages of change, according to the Prochaska and DiClemente’s Stages of Change model [33]
4. Change in the motivation to giving up smoking, according to the Richmond test [34]
5. Change in dependence, measured according to the Fagerström test [35]

## Methods/design

### Design and settings

This will be a single-centre prospective, randomized, controlled and open clinical trial with parallel groups set in the Nephrology Department at the Complexo Hospitalario Universitario A Coruña (northwest Spain). This is a 1382-bed public tertiary care hospital attending a population of nearly 560,000 habitants. The Nephrology department at Complexo Hospitalario Universitario de A Coruña is a reference hospital for renal transplantation at national level.

### Study period

Four years (from January 2012 to December 2015).

### Study population

**Fig. 1:**
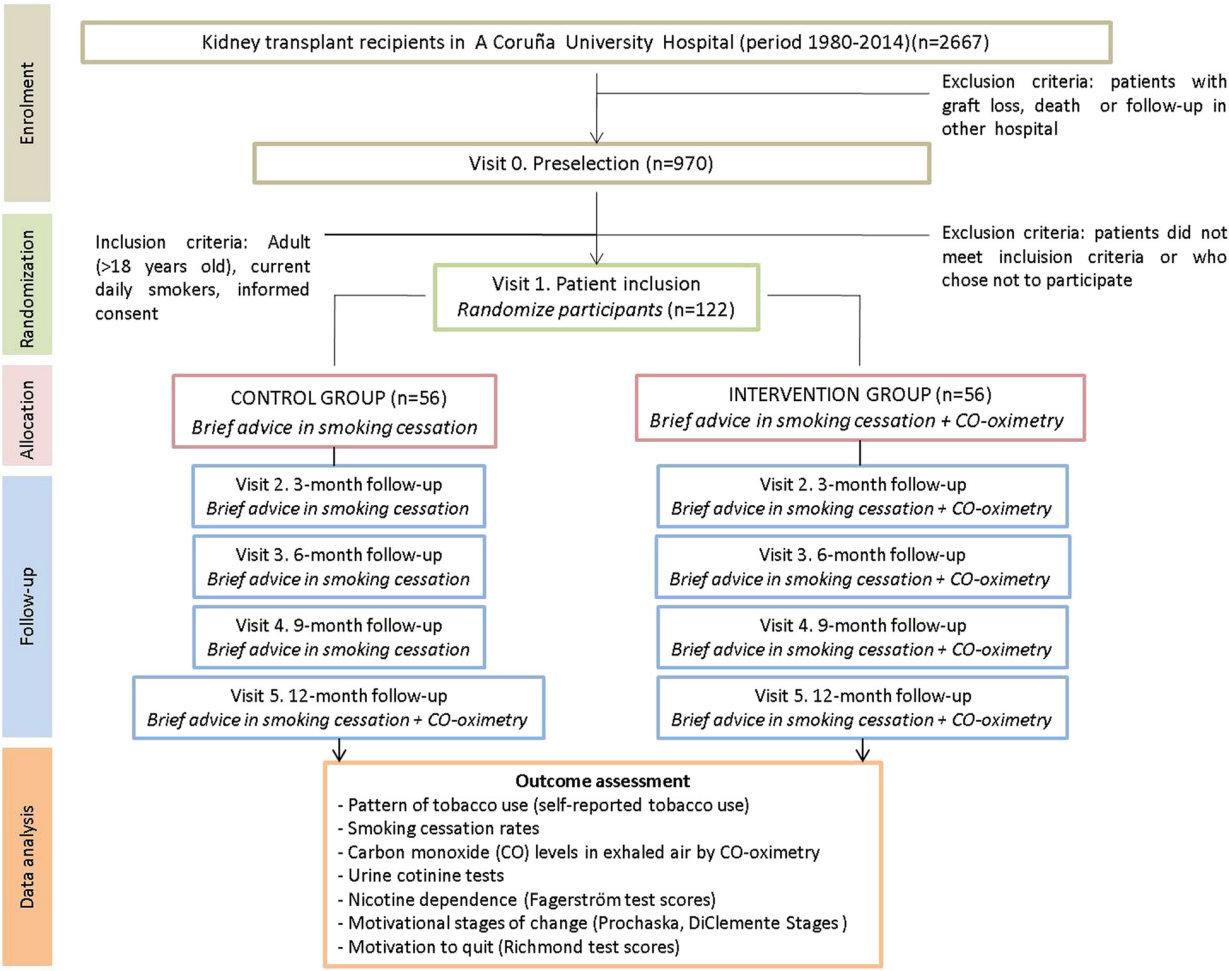
Flowchart of the study

All renal transplanted patients attending specialized consultations at the Nephrology department during the study period, who meet the inclusion criteria, are eligible to participate in the study. The flowchart of the study is shown in Fig. 1.

### Inclusion criteria

- Adult (over 18 years of age) kidney transplant recipients who smoke, with functioning allograft, who underwent primary or repeated renal transplantation from a cadaveric or a living donor in the A Coruña Hospital between 1981 and 2014
- Current daily smokers. Smoking status is defined according to the WHO classification. Therefore, a patient is considered a current daily smoker if they report daily smoking during the previous month, irrespective of the number of cigarettes smoked
- Patients in the precontemplation, contemplation or preparation stages, based on Prochaska and DiClemente’s transtheoretical model [33]

Smokers in the precontemplation stage have not seriously considered giving up smoking and do not think they will do so within the next 6 months. For the individual the pros of continuing smoking clearly outweigh the cons. They can be in this condition because they are misinformed or poorly informed about the consequences of their conduct or because they have tried to change it several times and are demoralized because they were not able to do so. They tend to avoid reading, speaking or thinking about the risk. Smokers in the contemplation stage have seriously considered giving up smoking within the next 6 months. They have not considered giving up smoking within the next 30 days or have not tried to stop for at least 24 h in the last year. They worry about the risks, their motivation to change or to remain without changing, and also their intention of changing in the long term, within the next 6 months but without specifying when. The smokers in the stage of preparation for the action have considered giving up smoking within the next 30 days, besides having made an attempt to abandon the habit for a duration of at least 24 h in the last year. Otherwise, they will be said to be in the stage of contemplation.

### Exclusion criteria

- Adult kidney transplant recipients whose grafts have failed
- Non-smoker and former smoker kidney transplant recipients
- Patients in the action stage on Prochaska and DiClemente’s transtheoretical model [33]
- Patients with any chronic or terminal condition that complicates the study interventions
- Patients with mental illness or transitory psychiatric deterioration at the moment of inclusion, who are not able to understand the study or complete the informed consent

### Recruitment of subjects

All renal transplanted patients will be contacted during their medical consultations at the hospital. They will be asked about their smoking habit. The patients who declare themselves smokers will be informed about the aims of the study and will be offered the chance to take part in it. An information sheet will be provided and informed consent will be obtained.

They will be asked about their consumption of tobacco and their attitude to giving up smoking with the aim of evaluating the phase of tobacco abandonment that they are on according to the process of change in Prochaska and DiClemente’s Stages of Change model [33].

Once the informed consent form has been signed each patient will be randomly assigned to the control group or the intervention group, according to the following scheme:

- Control group: brief advisory session to help give up smoking [36]
- Intervention group: brief advisory session on giving up smoking plus measurement of CO exhaled for CO-oximetry

### Sample size

Meta-analyses have shown that around 5–6 % of smokers stop after the brief advisory session from a physician [36]. A 13 % increase in the cessation rate for the brief advisory session plus CO-oximetry over the brief advisory session alone is considered clinically relevant. The sample target size is, therefore, 112 patients, with 56 patients in each group of the trial. Allowing for up to 10 % loss to follow-up, this would provide 80 % power to detect a 13 % difference in smoking cessation outcomes at a two-tailed significance level of 5 %.

### Blinding

This was an open clinical trial. Only those assessing outcomes were blinded after assignment to interventions.

### Random allocation to study arms

Computerized allocation to each of the study groups is done in advance. The assignment sequence is generated by a person who is not responsible for determining patient eligibility. By the nature of the interventions neither the researchers nor the patients can be blinded to the assignment.

### Intervention

At the baseline visit (visit 1) patients included in the study control group will be given a brief advisory session about giving up smoking. This will be firm, concise, personalized (trying to find the most important motivation for each patient) and appropriate to the phase towards cessation they are in. This will provide information about the negative effects of tobacco on their health and explain the main advantages of stopping smoking [36].

Patients included in the intervention group will receive the brief advisory session, as with the patients who belong to the control group, and in addition, CO-oximetry will be administered to them. This exploration allows one to know the amount of CO that the subject has in their body. The patient will take a deep breath and hold it for 15 s, then a slow, long and complete exhalation will be made. After a few seconds the oximeter indicator becomes stable and marks the exact number of particles per million (ppm) of CO that the subject has in the air they breathe. Follow-up visits will take place at 3 months (visit 2), 6 months (visit 3) and 9 months (visit 4). At each of these moments the anti-smoking advice will be repeated in the control group and anti-smoking advice plus CO-oximetry repeated in the intervention group (Table 2). At the 12-month (visit 5) visit, the anti-smoking advice will be given to both groups as well as CO-oximetry.

### Independent measures

From each patient included in the study the following variables will be recorded (Table 1):

**Table 1:** Baseline and follow-up measurements

	Basal	3 months	6 months	9 months	12 months
Donor variables	x				
Age (years)					
Gender					
Type of donor (deceased versus living)					
Recipient variables	x				
Age (years)					
Gender					
Chronic kidney disease-related risk factors	x				
Primary kidney disease					
Renal replacement therapy before transplantation					
Duration of renal replacement therapy before transplantation					
Cold ischemia time					
Pre-transplant cardiovascular risk factors	x				
Weight (kg), height (m) and body mass index (kg/m^2^)					
Pre-transplant systolic and diastolic blood pressure (BP)					
Pre-transplant cholesterol (mg/dl), high-density lipoprotein (HDL) cholesterol (mg/dl), low-density lipoprotein (LDL) cholesterol (mg/dl), and triglycerides (mg/dl)					
Cardiovascular events before transplantation					
Previous malignancies					
Left ventricular hypertrophy					
Post-transplant cardiovascular risk factors		x	x	x	x
Body mass index (kg/m^2^)					
Post-transplant systolic and diastolic blood pressure (BP)					
New-onset diabetes mellitus after transplantation					
Post-transplant left ventricular hypertrophy					
Routine biochemistry at follow-up	x	x	x	x	x
Creatinine (mg/dl), proteinuria (g/day), leukocyte count (number/l), hematocrit (%), hemoglobin (g/dl), serum albumin (g/dl), total cholesterol (mg/dl), HDL cholesterol (mg/dl), LDL cholesterol (mg/dl), and triglycerides (mg/dl)					
Follow-up of kidney transplantation		x	x	x	x
Post-transplant cardiovascular events					
Acute rejection episodes					
Graft failure					
Infections

1. Donor characteristics: age (years), gender and type of donor (deceased versus alive)
2. Recipient features:
  Sociodemographic variables: age (years), gender
  Chronic kidney disease-related risk factors: primary kidney disease, current treatment, previous transplants, cold ischemia time
  Pre-transplant cardiovascular risk factors: weight (kg), height (m) and body mass index (kg/m^2^), basal cholesterol, systolic and diastolic pre-transplant blood pressure. Hypertension is defined as blood pressure above 140/90 mmHg. Pre-transplant hypercholesterolemia is considered as a cholesterol value above 200 mg/dl. Cardiovascular events before the transplant, tumors prior to transplant, smoking exposure prior to transplant, pre-transplant diabetes mellitus, and left ventricular hypertrophy will also be recorded
  Post-transplant cardiovascular risk factors: body mass index (kg/m^2^), systolic and diastolic blood pressure after transplantation, new appearance of diabetes mellitus after transplantation, left ventricular hypertrophy after transplant
  Routine biochemistry on follow-up: creatinine (mg/dl), proteinuria (g/day), leukocyte count (number/l), hematocrit (%), hemoglobin (g/dl), serum albumen (g/dl), triglycerides (mg/dl), total cholesterol (mg/dl), HDL cholesterol (mg/dL), and LDL cholesterol (mg/dl)
  Transplantation outcomes in the follow-up: post-transplant cardiovascular events, acute rejection episodes, graft failure, and infections
3. Evaluation of use of, and exposure, to tobacco:
  At the time of their incorporation in the study, the following information will be collected from all the patients:
  Self-reported smoking habit: number of cigarettes smoked per day and number of years of being smoker (the pack-years index will be computed)
  Number of previous attempts to stop smoking
  Maximum period of time elapsed without smoking
  Consumption of tobacco between their family and/or circle of friends
  Both at baseline and during follow-up at 3, 6, 9 and 12 months, the following information will be collected from each patient:
  Self-reported use of tobacco and number of cigarettes smoked per day
  Urinary cotinine test
  Stage of change, according to the Prochaska and DiClemente’s Stages of Change model (precontemplation, contemplation, preparation, abandonment) [33]
  Nicotine dependence measured by the Fagerström test [35]. This test is used to evaluate the degree of physical dependence on nicotine. It consists of six items with two or four possible answers. The score ranges from 0 to 10. The cutoff points are 4 and 7, where less than 4 means a low dependence, between 4 and 7 means a moderate dependence, and over 7 means high dependence
  Motivation for giving up smoking, measured by the Richmond test [34]. This is a test that has four items with two or four possible answers. The score range is between 0 and 10, where item 1 rates between 0 and 1 and the other items range from 0 to 3.
    Based on this score, patients can be classified, according to their motivation for giving up smoking, in four groups: nil or low (0–3 points), doubtful (4–5 points), moderate (6–7 points), or high (8–10 points)

The results of the CO-oximetry in the intervention group will be collected at baseline, at 3 months, 6 months, 9 months and 12 months (visits 1–5). In the control group, CO-oximetry will only be performed at 12 months (visit 5).

### Outcome assessment

The effectiveness of both interventions will be evaluated at 3, 6, 9 and 12 months after inclusion in the study. The following outcomes will be investigated (Table 2):

**Table 2:** Interventions and measurements in the follow-up after randomization

	Basal	3 months	6 months	9 months	12 months
Group allocation and intervention					
, Control group:					
Brief advice on smoking cessation	x	x	x	x	x
, Intervention group:					
Brief advice on smoking cessation + CO-oximetry	x	x	x	x	x
Outcome assessment					
Pattern of tobacco use (self-reported tobacco use)	x	x	x	x	x
Smoking cessation rates		x	x	x	x
Carbon monoxide (CO) levels in exhaled air by CO-oximetry in the intervention group	x	x	x	x	x
Carbon monoxide (CO) levels in exhaled air by CO-oximetry in the control group					x
Urinary cotinine tests	x	x	x	x	x
Nicotine dependence (Fagerström test scores)	x	x	x	x	x
Motivational stages of change (Prochaska and DiClemente’s stages)	x	x	x	x	x
Motivation for giving up smoking (Richmond test scores)	x	x	x	x	x

1. Smoking habit cessation, confirmed by a urinary cotinine test. The intervention will be considered effective if the test results are lower than 100 ng/ml
2. Self-declared abandonment of smoking habit
3. Reduction in the number of cigarettes smoked per day self-declared by the patient
4. Variation in the motivational stage of change, according to the Prochaska and DiClemente’s Stages of Change model [33]
5. Change in motivation to giving up smoking, according to the Richmond test [34]
6. Change in nicotine dependence, measured according to the Fagerström test [35]

In the intervention group, the variation in CO levels in exhaled air, measured by CO-oximetry, will be registered. Main outcome will be mean urinary cotinine test differences between the two groups at 12 months. Secondary outcomes will include: urinary cotinine test results at 3, 6 and 9 months, CO-level differences at 12 months, as well as differences in self-reported tobacco use, nicotine dependence, Prochaska and DiClemente’s motivational stages of change and the Richmond test scores at 3, 6, 9 and 12 months of follow-up.

### Statistical analysis

Comparability of intervention and control groups will be checked in terms of the similarity of the distribution of the variables of interest at baseline. The response that patients rate, at different time points in the follow-up, will be compared in both arms of the study according to the outcomes studied.

The chi-square test or Fisher’s exact test will be used to compare proportions. Student’s *t* test will be used to compare means between groups with a normal distribution data. The Mann-Whitney test will be used to compare quantitative variables between groups in case of a non-normal distribution, determined by the Kolmogorov-Smirnov test.

Correlations between quantitative measurements will be determined by the Spearman’s rho correlation coefficient. Matched-pair data analysis will be also computed. Therefore, to evaluate the differences within each group at different time points, McNemar’s test and Wilcoxon’s signed ranks test will be calculated.

Additionally, clinical relevance from intervention will be studied by calculating the relative risk (RR), relative risk reduction (RRR), absolute risk reduction (ARR) and patient number needed to treat (NNT) at different times during the follow-up. All these measures will be presented with their confidence interval at 95 %.

A multivariate analysis using multiple linear regression and logistic regression will be performed, according to the considered response, to adjust the effectiveness of the intervention as potential confounders and to determine which other variables are associated with each of the results. Variables with statistical significance *p* <0.10 in the bivariate analysis will be selected to be included in the multivariate regression analysis. A modeling strategy of successive steps back will be used.

The degree of agreement between the self-declared smoking consumption, CO-oximetry results and the test results of urinary cotinine levels at 3, 6, 9 and 12 months after surgery will be evaluated. The agreement will be evaluated by the kappa index.

The validity of self-declared smoking consumption by patients regarding the results of the CO-oximetry and urinary cotinine test will be studied. Sensitivity, specificity and positive and negative predictive values will be determined, together with their 95 % confidence interval.

All analyses will be performed by intention-to-treat, in which the total value of randomization will be preserved and control over confounding reference will be also ensured. Analyses will be performed using the statistical package Statistical Package for the Social Sciences software, version 19.0 (SPSS, Chicago, IL, USA).

### Ethics

Investigators will ensure that this study is conducted in accordance with the principles of the Declaration of Helsinki and ICH Guidelines for Good Clinical Practice.

All participants will be informed of the study and informed consent will be obtained from all participants.

Participant confidentiality will be ensured. The study will comply with the Data Protection Legislation requirements for anonymization of data.

The study has received written approval from the regional Ethics Committee for Clinical Research (Comité Autonómico de Ética da Investigación de Galicia, EC registry code 2011/061).

All patients were required to give informed consent to participate in the clinical trial.

## Discussion

According to data published by OBLIKUE the total cost of a transplant in the first year is €47,136.33. This surgery is cheaper than dialysis and also gives the patient a higher likelihood of survival and better quality of life [37].

However, the transplanted kidney has to be cared for. Knowledge about risk factors for renal graft loss and its implications on morbidity and mortality are essential in trying to prevent this situation which can significantly harm the results that can be achieved with a kidney transplant.

In addition to the non-modifiable risk factors that have an influence on morbidity and mortality, there are others modifiables, such as tobacco smoking, whose identification and control is essential to help improve graft survival as tobacco smoking is a risk factor that causes cardiovascular disease and cancer [1] in renal transplant patients and tobacco consumption also negatively affects renal function [9, 10].

According to a previous study by our group, patients who continue smoking after kidney transplantation increase their risk of cardiovascular events compared to non-smokers and the incidence of such events also increases with time of exposure [12].

Of all the interventions that can be carried out, encouragement to create a habit of healthy living and the abandonment of smoking is the simplest and least expensive option and can be achieved by using a brief advisory session and conducting CO-oximetry.

Our intervention is aimed at kidney transplant patients for whom smoking is an added risk factor for impaired renal function.

Providing direct information related to health effects, adapted to each individual, and taking into account the stage of moving towards smoking abandonment that they are at helps make reduction of tobacco consumption possible. But performing CO-oximetry, a test in which the patient can actually see a number and a color that tells them whether they have improved compared to previous visits, has proved to be more effective in helping successful smoking cessation [38, 39].

In relation to self-reported consumption of tobacco the validity is questioned because of the belief that smokers tend to underestimate the amount they smoke [40–43].

As a result of misclassification or deception regarding self-reports of smoking status, due to information bias, biochemical measures are recommended to validate self-reports of smoking behavior among patients who are participants in evaluation studies [44–46].

Moreover, on the other hand this study will determine the correlation between self-reported exposure and cotinine findings using a urine test strip as well as those from CO-oximetry. Although CO measurement is not the best indicator of smoking cessation, at least for occasional smokers, due to its short half-life, the use of different outcome measures at different moments of time will allow us to see the consistency of the results among them.

Finally, if effectiveness of CO-oximetry is confirmed to reduce tobacco exposure in this subset of patients, we would have an intervention that is easy to use, economic and with considerable implications for cardiovascular risk prevention.

### Trial status

At the time of submission, this trial is in the process of participant recruitment.

## Abbreviations

CO: carbon monoxide
HDL: high-density lipoprotein
LDL: low-density lipoprotein
NHS: National Health Survey
NNT: number needed to treat
ppm: particles per million
ARR: absolute risk reduction
RR: relative risk
RRR: relative risk reduction
WHO: World Health Organization
